# Protecting effects of 4-octyl itaconate on neonatal hypoxic-ischemic encephalopathy via Nrf2 pathway in astrocytes

**DOI:** 10.1186/s12974-024-03121-8

**Published:** 2024-05-17

**Authors:** Yanping Yang, Yang Li, Wenyi Yang, Xueying Yang, Man Luo, Ling Qin, Junchao Zhu

**Affiliations:** 1 Department of Anesthesiology, The Shengjing Hospital of China Medical University, Shenyang, Liaoning China; 2 Department of Physiology, China Medical University, Shenyang, Liaoning China; 3 Department of Anesthesiology, Shenzhen Cancer Hospital, Shenzhen, China

**Keywords:** Hypoxia–ischemia, Itaconate, Nrf2, Astrocyte, Cognitive dysfunction

## Abstract

**Background:**

Neonatal hypoxic-ischemic encephalopathy (HIE) is one of the most common neurological problems occurring in the perinatal period. However, there still is not a promising approach to reduce long-term neurodevelopmental outcomes of HIE. Recently, itaconate has been found to exhibit anti-oxidative and anti-inflammatory effects. However, the therapeutic efficacy of itaconate in HIE remains inconclusive. Therefore, this study attempts to explore the pathophysiological mechanisms of oxidative stress and inflammatory responses in HIE as well as the potential therapeutic role of a derivative of itaconate, 4-octyl itaconate (4OI).

**Methods:**

We used 7-day-old mice to induce hypoxic-ischemic (HI) model by right common carotid artery ligation followed by 1 h of hypoxia. Behavioral experiments including the Y-maze and novel object recognition test were performed on HI mice at P60 to evaluate long-term neurodevelopmental outcomes. We employed an approach combining non-targeted metabolomics with transcriptomics to screen alterations in metabolic profiles and gene expression in the hippocampal tissue of the mice at 8 h after hypoxia. Immunofluorescence staining and RT-PCR were used to evaluate the pathological changes in brain tissue cells and the expression of mRNA and proteins. 4OI was intraperitoneally injected into HI model mice to assess its anti-inflammatory and antioxidant effects. BV2 and C8D1A cells were cultured in vitro to study the effect of 4OI on the expression and nuclear translocation of Nrf2. We also used Nrf2-siRNA to further validate 4OI-induced Nrf2 pathway in astrocytes.

**Results:**

We found that in the acute phase of HI, there was an accumulation of pyruvate and lactate in the hippocampal tissue, accompanied by oxidative stress and pro-inflammatory, as well as increased expression of antioxidative stress and anti-inflammatory genes. Treatment of 4OI could inhibit activation and proliferation of microglial cells and astrocytes, reduce neuronal death and relieve cognitive dysfunction in HI mice. Furthermore, 4OI enhanced nuclear factor erythroid-2-related factor (Nfe2l2; Nrf2) expression and nuclear translocation in astrocytes, reduced pro-inflammatory cytokine production, and increased antioxidant enzyme expression.

**Conclusion:**

Our study demonstrates that 4OI has a potential therapeutic effect on neuronal damage and cognitive deficits in HIE, potentially through the modulation of inflammation and oxidative stress pathways by Nrf2 in astrocytes.

**Supplementary Information:**

The online version contains supplementary material available at 10.1186/s12974-024-03121-8.

## Introduction

Neonatal hypoxic-ischemic encephalopathy (HIE) is a brain injury caused by perinatal asphyxia in neonates, with its incidence from 1 to 8 per 1000 live births in developed countries and reaching as high as 26 per 1000 live births in underdeveloped countries [1–3]. Currently, therapeutic hypothermia (TH) is the only intervention approved for the treatment of HIE [4]. However, its application is constrained by a narrow therapeutic window, requiring initiation within six hours post-birth. Despite the widespread implementation of TH in high-resource settings, approximately 30–50% of infants with moderate-to-severe HIE treated with TH still suffer from mortality or substantial neurological impairments, such as cerebral palsy, epilepsy and cognitive dysfunction [5–10]. Therefore, it is imperative to explore new neuroprotective therapies to reduce the mortality rate of HIE patients and improve their neurological development outcomes.

During the initial stage of HIE pathophysiology (0–6 h), the brain undergoes a complex cascade of processes associated with energy depletion, including the accumulation of excitotoxic substances, oxidative stress, and intracellular acidosis [11–13]. Compared to the adult brain, the neonatal brain has a high level of mitochondrial respiration for oxygen consumption and low concentrations of antioxidants, rendering it particularly susceptible to the detrimental effects of oxidative stress [14, 15]. Previous studies have indicated that the hippocampus is more susceptible to oxidative stress damage induced by hypoxia–ischemia due to its high content of polyunsaturated fatty acids and abundance of transition metals such as iron and copper, leading to adverse cognitive effects [16–18]. Although studies have shown that the hippocampus is a brain region with neurogenesis and neurogenesis increases after HI injury, it is still impossible to fully compensate for the neurological impairment after HI [19, 20]. Therefore, it is imperative to study the pathophysiological and molecular mechanisms of HI damage to hippocampal neurons, in order to provide experimental evidence for the development of new therapeutic approaches.

Following HI, damaged neurons activate brain innate immune cells-microglia through multiple pathways such as damage-related molecular patterns (DAMPs) by releasing ADP, heat shock protein and other substances, prompting them to release inflammatory factors such as IL-1β, IL-6, and TNF-α, as well as reactive oxygen species, and induce neuronal damage and even death through pathways such as NF-κB [21]. Studies have shown that activated microglia release CSF-1, which promotes the transformation of local astrocytes from nutrient-supportive to reactive state, and induces the production of pro-inflammatory cytokines. This cross talk between microglia and astrocyte would lead to a persistent inflammatory response [22]. Oxidative stress-induced inflammatory responses, if persistent, result in widespread neuronal death and permanent neurological impairment in the hippocampus [23, 24].

Therefore, it is crucial to identify key molecular mechanisms of neuron-glial cross-talk induced by oxidative stress and inflammatory response in hippocampus. The results of this study can provide a basis for targeted therapy for cognitive dysfunction in HIE. To achieve this, In this study, we employed an approach combining non-targeted metabolomics with transcriptomics, to screen alterations in metabolic profiles and gene expression in the hippocampal tissue of a mouse model 8 h after hypoxia–ischemia (HI). We identified significant metabolic perturbations, including the accumulation of lactate and pyruvate. And the RNA-sequence (RNA-seq) results showed high expression of inflammatory genes related to IFN and nuclear factor (NF)- κB pathways, as well as elevated expression of antioxidant stress genes, such as activating transcription factor 3 (Atf3), the nuclear factor erythroid-2-related factor (Nfe2l2; Nrf2), and heme oxygenase 1 (Hmox-1;HO-1). The results of metabolomic and RNA-Seq indicate that pro-inflammatory and anti-inflammatory, oxidative and antioxidative pathways were simultaneously activated.

Based on these mechanistic insights, we speculate that potentiating Nrf2- mediated anti-inflammatory and antioxidative pathways may have a protective effect on HIE. Recently, some studies have shown that 4-octyl itaconate (4OI), a derivative of itaconate, could facilitate Nrf2 to translocate into the nucleus and upregulate the expression of anti-inflammatory and antioxidant genes [25–28]. In addition, potential therapeutic effects of itaconate has been confirmed in various central nervous system disorders, including Alzheimer's disease and spinal cord injury [29–32]. However, the role and mechanism of 4OI in HI remain unexplored. Therefore, we injected 4OI intraperitoneally into HI model mice, it was found that 4OI exerts neuroprotective effects in HI mice through increasing the level of Nrf2 in astrocytes, thereby suppressing the production of pro-inflammatory cytokines, such as interleukin (IL)-1β and IL-6, and regulating the expression of antioxidant enzymes HO-1. Our findings provide experimental evidence for the essential role of Nrf2 in the pathological process of HIE, and the potential for clinical applications of 4OI as a therapeutic drug to HIE.

## Materials and methods

### Animals

In this study, all procedures and protocols were approved by the Animal Care and Use Committee of China Medical University. Male and female C57BL/6 wild-type mice aged P7–P60 weeks were obtained from the Department of Laboratory Animal Science of China Medical University. The pups, along with their mothers, were bred and reared under standard conditions with a 12 h/12 h light/dark cycle, at an environmental temperature of 22 ± 1 °C and a humidity of 30%—50%, with ad libitum access to food and water. All surgical procedures were conducted under anesthesia to minimize animal suffering. To reduce experimental bias and ensure unbiased results, all animals were randomly assigned to groups generated by Excel. All researchers involved in neurological testing and molecular experiments were blinded. Each intervention group included an appropriate control group.

### Neonatal hypoxia–ischemia model and drug administration.

This study utilized the classic model of neonatal hypoxic-ischemic brain damage [33], conducting experiments in line with the animal ethics guidelines set by China Medical University. We used 3–5 g of mixed gender (randomized males and females) P7 pups for experiments. Initially, the pups were anesthetized with 2% isoflurane. Following anesthesia, their neck skin was disinfected with alcohol. Subsequently, a 1 cm midline incision was made on the right anterior neck using a scalpel. A gentle blunt dissection was then performed, delicately separating the right common carotid artery from surrounding structures. Right common carotid artery was carefully double ligated with 8–0 surgical silk and subsequently cut between the ligatures. After this, the skin was sutured with thread. To maintain sterility, the entire surgical process was conducted under aseptic conditions, ensuring that each surgery lasted no more than 10 min. Postoperatively, the pups were placed on a 37 °C heating blanket for 1 h for recovery. In our study, we modified a sealed mice anesthesia chamber, with one end connected to pure nitrogen and the other to pure oxygen. The oxygen concentration could be adjusted by altering the flow rate of oxygen or nitrogen through a gas valve. Additionally, the chamber was equipped with a CY-12 oxygen analyzer and a thermometer for real-time monitoring of oxygen concentration and temperature. The pups were placed in a temperature-controlled hypoxic chamber for 1 h (7% O_2_, 93% N_2_, at 37 °C). Finally, the pups were returned to their mother and monitored daily. In contrast, sham-operated mice only underwent a neck skin incision without any ligation or hypoxia. Due to the absence of a monitoring system for vital signs during the modeling process, there was an unavoidable mortality rate of approximately 5% among the neonatal mice.

In determining the dosage and duration for 4OI treatment, we referenced a recent review on the dosage of 4OI (PMID: 34055739), which provided a foundation for selecting the doses of 10, 25, and 50 mg/kg. Additionally, we decided to administer 4OI at the determined optimal dose every 24 h for a total of 3 days, based on studies indicating that delayed neuronal death in the hippocampus occurs 3–4 days following ischemia–reperfusion (PMID: 31251935).

### The OGD/R model

BV2 microglial cells and C8-D1A astrocytes were purchased from Procell (Wuhan, China). Both cell types were cultured in complete medium containing 10% fetal bovine serum for two days. At the onset of the oxygen–glucose deprivation (OGD) experiment, the culture medium was replaced with a glucose- and serum-free complete medium, and cells were placed in a hypoxic chamber (1% O_2_, 5%CO_2_, 94%N_2_, at 37 °C, Biospherix C21, American) for 4 h of hypoxia. Following the OGD/R, the medium was again replaced with serum-containing complete medium, and cells were further cultured for 12 h to complete the oxygen–glucose deprivation/reoxygenation (OGD/R) process. In contrast, control group cells were cultured in a standard incubator (5% CO_2_, 94% O_2_, at 37 °C) with complete medium. The experiment was divided into three groups: control (Control), OGD/R, and OGD/R + 4OI. In the control and OGD/R groups, DMSO was added to the culture medium to a final concentration of 0.2%. In the OGD/R + 4OI group, 4OI (50 μM, 0.1% DMSO) was dissolved in the culture medium. After the OGD treatment, cell supernatants were collected for subsequent analyses.

### In vitro cell transfection

Small interfering RNA (siRNA) targeting Nrf2 (si-Nrf2), and negative scramble control siRNA (si-Negative) were purchased from Thermo Fisher Scientific (USA). C8-D1A astrocytes were cultured in complete medium in 6-well plates for 48 h, and then transfected with either Nrf2 siRNA or Nrf2-negative siRNA using Lipofectamine RNAi MAX (Invitrogen, Waltham, MA, USA). After 24 h, the transfected cells were exposed to OGD/R for 4 h before Western blotting and further analysis.

### Western Blot

Western blotting was performed in accordance with the manufacturer's specifications. After OGD treatment, C8-D1A and BV2 cells were collected. Cells were lysed in RIPA buffer containing phenylmethylsulfonyl fluoride (Santa Cruz, CA, USA) for protein extraction, followed by centrifugation at 12,000 rpm for 15 min at 4 °C to remove insolubles. Protein concentration was quantified using the BCA Protein Assay Kit (Beyotime technology, China). Equal amounts of protein were loaded onto SDS-PAGE gels for electrophoresis, followed by transfer to polyvinylidene fluoride membranes. After blocking with 5% non-fat milk for 2 h, the membranes were incubated overnight at 4 °C with Nrf2, IL-1β (1:2000, 16,806–1-AP, Protrintech) and HO-1 (1:1000, 10,701–1-AP, Protrintech) antibody. After primary antibody incubation, membranes were washed at least three times with TBS containing 0.2% Tween-20, then incubated with the appropriate secondary antibody at room temperature for 1.5 h. The membranes were then washed three times with TBST. Finally, detection of protein bands was conducted using an enhanced chemiluminescence reagent and imaged with an imaging densitometer. Data analysis was performed using ImageJ software.

### TTC staining

The pups were fully anesthetized with isoflurane (3%) and perfused with PBS through the heart to collect the brain 24 h post-HI. The brain was then frozen in a -20℃ freezer for 20 min, and sliced into 3 mm coronal sections with a blade. A total of 4 coronal brain slices were prepared. The brain slices were incubated in 2% 2,3,5-triphenyltetrazolium chloride monohydrate (TTC) staining solution at 37℃ in the dark for 30 min, and then fixed with 4% paraformaldehyde [34]. The brain infarct volume of the mice was calculated using the Image-J software (version) by scanning the coronal brain tissue sections with a scanner (Hp laserJet Pro MFP M427dw). The formula used for calculating the infarct volume (%) was as follows: Infarct volume (%) = [Volume of the contralateral hemisphere—(Volume of the ipsilateral hemisphere—infarct volume)] / Volume of the contralateral hemisphere × 100% [35].

### Evaluation of brain tissue loss

The percentage of brain tissue loss = (contralateral hemisphere – ipsilateral hemisphere)/contralateral hemisphere × 100% [36].

### Immunofluorescence staining

The mice were anesthetized at P8, P14 and P60, and then perfused transcardially with ice-cold PBS and 4% paraformaldehyde. The brains were fixed with 4% paraformaldehyde for 24 h and transferred to 30% sucrose solution for dehydration for three days. The brain tissues were sliced continuously at -20 °C using a freezing microtome (Minux, FS800, RWD) to obtain coronal sections of 15 μm-thick coronal sections. The sectioned tissue was then fixed on glass slides and used for immunofluorescence staining. The brain slices were washed three times with PBS solution (5 min per wash), blocked with 10% goat serum for 2 h, and then incubated with primary antibodies, including Anti-Neun (1:500, ab177487, Abcam, USA), Anti-GFAP (1:500, ab7260, Abcam, USA), Anti-Iba1 (1:200, ab178847, Abcam, USA), and Anti-Nrf2 (1:50, 1639–1-AP, Proteintech, USA), at 4 °C for 12 h. The slices were washed three times with PBS solution (5 min per wash) and incubated with secondary antibodies, including CoraLite488 (1:200, SA00013-2, Proteintech, USA) and CoraLite594 (1:200, SA00013-4, Proteintech, USA), at room temperature for 2 h. After washing with PBS, sections were covered with DAPI (SL1841, Coolaber, China). Finally, the slides were observed using a fluorescence microscope (BX53, Olympus, Japan), and the number of cells in the hippocampus were automatically identified and counted using IMARIS software (Bitplane, Switzerland).

### RNA extraction, library preparation and sequencing

At P7, mice were deeply anesthetized with 3% isoflurane and were perfused transcardially with 100 mL 0.9% NaCl (4 °C). After the perfusion, hippocampal tissues from the side corresponding to the ligated right common carotid artery were harvested and immediately immediately preserved in the RNA later solution. Total RNAs were extracted from hippocampus of sham and HI mice using TRIzol Reagent (Invitrogen, cat. NO 15596026) following the methods by Chomczynski et al.[37]. DNA digestion was carried out after RNA extraction by DNaseI. RNA quality was determined by Nanodrop TM OneC spectrophotometer (Thermo Fisher Scientific Inc). RNA Integrity was confirmed by 1.5% agarose gel electrophoresis. Qualified RNAs were finally quantified by Qubit3.0 with QubitTM RNA Broad Range Assay kit (Life Technologies, Q10210).

2 μg total RNAs were used for stranded RNA sequencing library preparation using KC-DigitalTM Stranded mRNA Library Prep Kit for Illumina® (Catalog NO. DR08502, Wuhan Seqhealth Co., Ltd. China) following the manufacturer’s instruction. The kit eliminates duplication bias in PCR and sequencing steps, by using unique molecular identifier (UMI) of 8 random bases to label the pre-amplified cDNA molecules. The library products corresponding to 200–500 bps were enriched, quantified and finally sequenced on DNBSEQ-T7 sequencer (MGI Tech Co., Ltd. China) with PE150 model.

### RNA-Seq data analysis

Raw sequencing data was first filtered by Trimmomatic (version 0.36), low-quality reads were discarded and the reads contaminated with adaptor sequences were trimmed. Clean Reads were further treated with in-house scripts to eliminate duplication bias introduced in library preparation and sequencing. In brief, clean reads were first clustered according to the UMI sequences, in which reads with the same UMI sequence were grouped into the same cluster. Reads in the same cluster were compared to each other by pairwise alignment, and then reads with sequence identity over 95% were extracted to a new sub-cluster. After all sub-clusters were generated, multiple sequence alignment was performed to get one consensus sequence for each sub-clusters. After these steps, any errors and biases introduced by PCR amplification or sequencing were eliminated.

Reads mapped to the exon regions of each gene were counted by featureCounts (Subread-1.5.1; Bioconductor) and then RPKM was calculated. Genes differentially expressed between groups were identified using the edgeR package (version 3.12.1). A p-value cutoff of 0.05 and Fold-change cutoff of 2 were used to judge the statistical significance of gene expression differences. Gene ontology (GO) analysis and Kyoto encyclopedia of genes and genomes (KEGG) enrichment analysis for differentially expressed genes were both implemented by KOBAS software (version: 2.1.1) with a P-value cutoff of 0.05 to judge statistically significant enrichment. Alternative splicing events were detected by using rMATS (version 3.2.5) with a FDR value cutoff of 0.05 and an absolute value of Δψ of 0.05.

### Sample preparation and extraction

The sham and HI hippocampal sample stored at -80 °C refrigerator was thawed on ice and vortexed for 10 s. 50 μL of sample and 300 μL of extraction solution (ACN: Methanol = 1:4, V/V) containing internal standards were added into a 2 mL microcentrifugetube. A 180 μL aliquots of supernatant were transferred for LC–MS analysis. The original data file acquisited by LC–MS was converted into mzML format by ProteoWizard software. Peak extraction, peak alignment and retention time correction were respectively performed by XCMS program. The “SVR” method was used to correct the peak area. The peaks with detetion rate lower than 50% in each group of samples were discarded. After that, metabolic identification information was obtained by searching the laboratory’s self-built database, integrated public database, AI database and metDNA.

### Metabolomics analysis

Unsupervised PCA (principal component analysis) was performed by statistics function prcomp within R(www.r-project.org). The data was unit variance scaled before unsupervised PCA. The HCA (hierarchical cluster analysis) results of samples and metabolites were presented as heatmaps with dendrograms, while pearson correlation coefficients (PCC) between samples were caculated by the cor function in R and presented as only heatmaps. Both HCA and PCC were carried out by R package ComplexHeatmap. For HCA, normalized signal intensities of metabolites (unit variance scaling) are visualized as a color spectrum. For two-group analysis, differential metabolites were determined by variable importance on projection (VIP) value > 1 and P-value (P-value < 0.05, Student’s t test). VIP values were extracted from OPLS-DA result, which also contain score plots and permutation plots, was generated using R package MetaboAnalystR. The data was log transform (log2) and mean centering before OPLS-DA. In order to avoid overfitting, a permutation test (200 permutations) was performed. Identified metabolites were annotated using KEGG Compound database (http://www.kegg.jp/kegg/compound/), annotated metabolites were then mapped to KEGG Pathway database (http://www.kegg.jp/kegg/pathway.html). Significantly enriched pathways are identified with a hypergeometric test’s P-value for a given list of metabolites.

### Real-time PCR

After completely anesthetizing the mice with isoflurane, the hippocampal tissue on the same side was collected. The RNA was extracted using an RNA Isolation Kit-BOX2 (RC101-01, Vazyme, China) following the manufacturer's instructions, and its concentration and quality were measured using a fluorescence method. Reverse transcription was performed using a cDNA synthesis kit (TaKaRa Biotechnology). IL-1β and IL-6 primers (Sangon Biotech, Shanghai, China) were used for qPCR on a SYBR-Green pre-mix (TransGen Biotech). The cycle parameters of the CFX96 sequence detection system were 95 °C for 5 min, followed by 40 cycles of 95 °C for 15 s, 58 °C for 30 s, and 72 °C for 20 s. Finally, the mRNA level of β-actin was used as an internal control to standardize the expression level of the target gene. The ΔΔCt values of each group were analyzed, and the mRNA expression of different groups was normalized to 2-ΔΔCt (Table 1).

**Table 1:** Primers used in this study

Primer	Forward sequence (5’-3’)	Reverse sequence (5’-3’)
IL-1β	CGTCTCCCAGAGCCAATCC	CACCAGGCTGACTTTGAGTGAGT
IL-6	ATCCAGTTGCCTTCTTGGGACTGA	TAAGCCTCCGACTTGTGAAGTGGT
C3	TTCCTTCACTATGGGACCAGC	TCCTTACTGGCTGGAATCTTGA
Ctla2a	GGTGAGAGCAGAGAAAAGCA	CTGTGTCTTTCTTCATTCAGATTGT
Ifi211	AGCTGATTCTGGATTGGGCA	TCACACACTTTCTGCGTGCT
Nfe2l2	ACTACAGTCCCAGCAGAGTGAT	TCACACACTTTCTGCGTGCT
Atf3	TACCGTCAACAACAGACCCC	CCAGTTTCTCTGACTCTTTCTGC
HO-1	CCGCTACCTGGGTGACCTCTC	CCTCTGACGAAGTGACGCCATC
β-actin	TAAAGACCTCTATGCCAACACAGT	CACGATGGAGGGGCCGGACTCATC

*IL* interleukin, *C3* complement component 3, *Ctla2a* cytotoxic T lymphocyte-associated protein 2 alpha, *Ifi211* interferon activated gene 211, *Nfe2l2* nuclear factor, erythroid derived 2, like 2, *Atf3* activating transcription factor 3, *HO-1* heme oxygenase 1

### Behavior tests

#### Negative geotaxis

Set the wooden board at a 45-degree angle and release the mouse with its head facing the lower end of the slope in the middle of the board. Record the time it takes for the mouse to turn its head and body 180 degrees, repeating the experiment three times per mouse and calculating the average. If the mouse does not turn its head within 60 s, the maximum value is recorded as 60 s.

#### Righting reflex test

Place the young mouse with its back facing upwards on a wooden board, ensuring that its head and back are tightly against the board before releasing it. Subsequently, record the time it takes for the young mouse to turn from a supine position to a prone position with its limbs facing the board. Repeat this process three times for each mouse and calculate the average time. If a mouse fails to turn over within 60 s, record the time as the maximum value of 60 s.

#### Y-maze

The Y-maze consists of three arms, each measuring 10 cm × 40 cm × 15 cm, with an angle of 120° between each arm. The entire Y-maze experiment consists of two phases. In the first phase, one arm of the Y-maze (the novel arm) is randomly blocked using an opaque barrier, and the mouse is placed randomly in one of the remaining two arms (the start arm) for free exploration for 5 min. Afterwards, the mouse is returned to the home cage. The Y-maze is cleaned of feces and wiped with 75% ethanol to eliminate any olfactory cues that may affect the mouse. The second phase of the experiment begins 5 min later, during which the opaque barrier is removed, and the mouse is placed back in the same start arm for free exploration for 5 min while being monitored by a camera. A custom-built software is used to track the mouse's location and analyze its trajectory after the experiment. The exploration time of the novel arm is recorded, and the percentage of time spent exploring the novel arm is calculated as a proportion of the total exploration time.

#### Novel object recognition (NORT)

The novel object recognition test was used to detect short-term memory function. The experiment consists of three phases: habituation, training, and testing. During the habituation phase, the mouse is allowed to adapt to a black and white box (50 cm × 40 cm × 30 cm) for 30 min. The training phase begins 24 h later, during which the mouse is placed in the box with two identical objects for free exploration for 10 min, followed by returning the mouse to its home cage. The Y-maze is cleaned of feces and wiped with 75% ethanol to eliminate any olfactory cues that may affect the mouse. The testing phase starts by replacing one object with a novel object, and the mouse is allowed to explore for another 10 min. The mouse's movement trajectory is recorded and analyzed by a custom-built software to quantify the exploration time on different objects and evaluate the cognitive function of the mouse using the novel object recognition test index. The discrimination index (DI) is calculated as follows: DI = [(novel object exploration time)—(familiar object exploration time)] / [(novel object exploration time) + (familiar object exploration time)], where DI represents the recognition ability of the mouse [38].

#### The open field test (OFT)

The open field apparatus consisted of a rectangular behavioral box made of white plastic panels (54 cm × 40 cm), with a height of 30 cm. Prior to the initiation of the experiment, each mouse was placed in the laboratory for 5 min in advance to acclimate to the laboratory environment. At the start of the experiment, mice were placed in the central area of the behavioral box and allowed to freely move and explore the entire box for 30 min. Their activities were recorded by a camera (C270 HD Webcam, Logitech, Switzerland) positioned approximately 80 cm above the box. Following the test, mice were removed from the behavioral box. Each animal was subjected to the open field test only once. Between each experiment, excrement within the box was cleaned, and the box was thoroughly disinfected with 75% ethanol to eliminate the impact of residual biological odors on the experiment. Results include total distance traveled and duration in center (located in the center of arena with 25% of total area). The total duration of OFT, excluding habituation, was 30-min.

#### The forced swimming test

The forced swim test is conducted in a cylindrical container filled with water, typically 30 cm in diameter and 45 cm in height. The container is filled with water maintained at 22–25 °C, with a depth sufficient to prevent the mouse from touching the bottom with its feet or tail while preventing escape. Each mouse is gently placed into the water and allowed to swim for a fixed duration, commonly 6 min. The initial 2 min serve as an acclimation period, and the subsequent 4 min are recorded for analysis. The swim behaviors of the mice are captured by a camera positioned above the container. Each mouse undergoes the forced swim test only once to prevent the influence of repeated testing. Following the test, mice are carefully removed from the water, dried, and returned to their home cages. The water in the container is changed between each test to maintain cleanliness and consistent testing conditions. Immobility time was defined as the time spent by the mouse floating in the water without struggling and making only those movements necessary to keep its head above the water.

#### The elevated O-maze experiment

The elevated O-maze is a device developed from the elevated plus maze, designed to enable continuous exploration by experimental animals. The elevated O-maze consists of an elevated circular platform with an external diameter of 50 cm and an internal diameter of 30 cm. It is comprised of two opposite closed arms (10 cm wide and 12 cm high) and two opposite open arms (10 cm wide), set at a height of 100 cm above the ground. Each mouse was given 5 min to acclimate to the laboratory environment before being gently lifted by the tail and placed in an open arm of the elevated O-maze, where it was allowed to explore for 10 min. The activity of the mice in the elevated O-maze was captured by an overhead camera (C270 HD Webcam, Logitech, Switzerland). Each experimental animal was subjected to the elevated O-maze test only once. The entire elevated O-maze platform was cleaned with 75% ethanol between each experiment. The time spent in the open arms was used as an index of anxiety-like behavior.

### Statistical analysis

Statistical analysis in this study was performed using GraphPad Prism 8 software (GraphPad Software, San Diego, USA). The data were presented as mean ± standard error of the mean. Student's t-test was used to analyze significant differences between the two groups, and one- or two-way ANOVA was used to analyze the differences more than two groups, followed by Tukey's post-hoc or Sidak multivariate comparative analysis. A significance level of p < 0.05 was considered statistically significant.

## Results

### Cognitive dysfunction in adult HI model mice

**Fig. 1:**
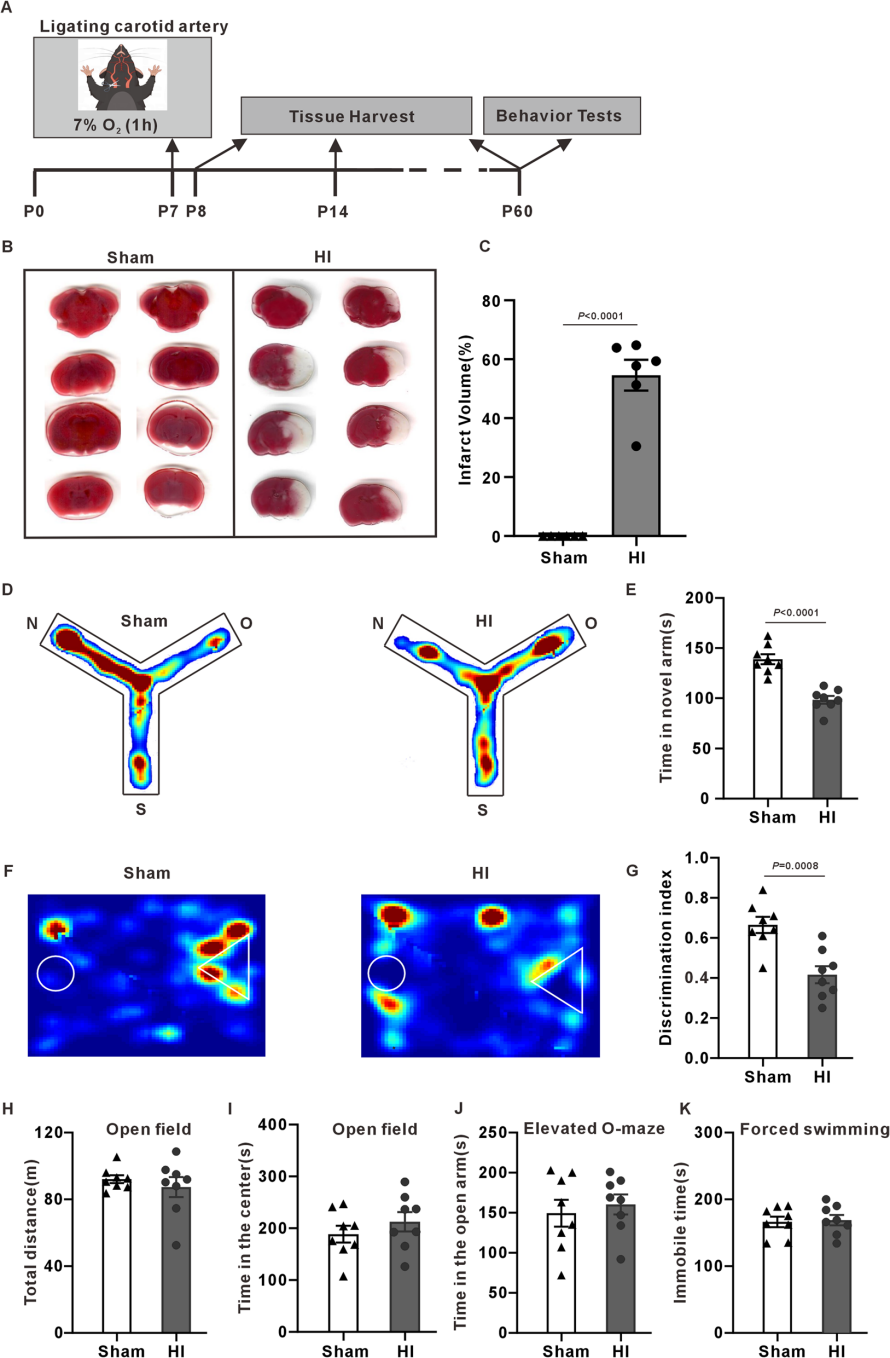
Assessment of the degree of brain damage and behavioral changes in HI mice. **A** Flow chart of HI modeling and experimental design. Mice are ligated with the right common carotid artery at P7 postnatal, then hypoxia for 1 h (7% O2), tissue harvest is performed at P8, P14 and P60, and behavioral tests are conducted at P60. **B** Representative images of TTC-stained coronal brain sections from sham and HI mice 24 h post-HI. The red areas indicate the reaction of TTC reagent with mitochondrial respiratory enzymes in living tissues, while the white areas represent infarcted tissue. Notably, large cerebral infarctions are observed in the HI group. **C** Mean and standard error of the infarct volume (two-tailed unpaired Student’s t-test, n = 6 mice/group). **D** Representative trace track of mice in Y-maze test. Start arm(S), Old arm(O), New arm(N). **E** Time to explore within the new arms (two-tailed unpaired Student’s t-test, n = 8 mice/group). **F** Representative trace track of mice in the novel object recognition test. **G** Discrimination index (two-tailed unpaired Student’s t-test, n = 8 mice/group). **H**, **I** Total distance and center exploration time in the open field experiment (two-tailed unpaired Student’s t-test, n = 8 mice/group). **J** Immobile time in the forced swimming (two-tailed unpaired Student’s t-test, n = 8 mice/group). **K** Time in the open arm of the elevated O-maze (two-tailed unpaired Student’s t-test, n = 8 mice/group)

As shown in Fig. 1A, we used HI on 7-day-old mice as an experimental model, which was the modified Rice-Vannucci neonatal hypoxia–ischemia model to ensure the survival rate of pups [39]. The results of TTC staining showed that there were no white infarcts in the sham group. In the HI group, large white infarcts appeared in the right cerebral hemisphere, and the average infarct volume accounted for 55.08% ± 5.40% of the whole brain volume (Fig. 1B, C). We performed several behavioral tests on HI mice at P60 stage to evaluate long-term outcomes. Compared with the sham group, HI mice exhibited less time during exploration in the novel arm of the Y-maze (Fig. 1D, E). The novel object recognition test revealed that the discrimination index was significantly lower in the HI group than in the sham group (Fig. 1F, G), indicating the object recognition memory and the short-term memory were impaired. Besides, no significant difference was found in open field, elevated O-maze and forced swimming tests (Fig. 1H–K). Our results showed that HI caused cognitive dysfunction in mice.

### Pathological Changes in the Hippocampus of Mice after HI

**Fig. 2:**
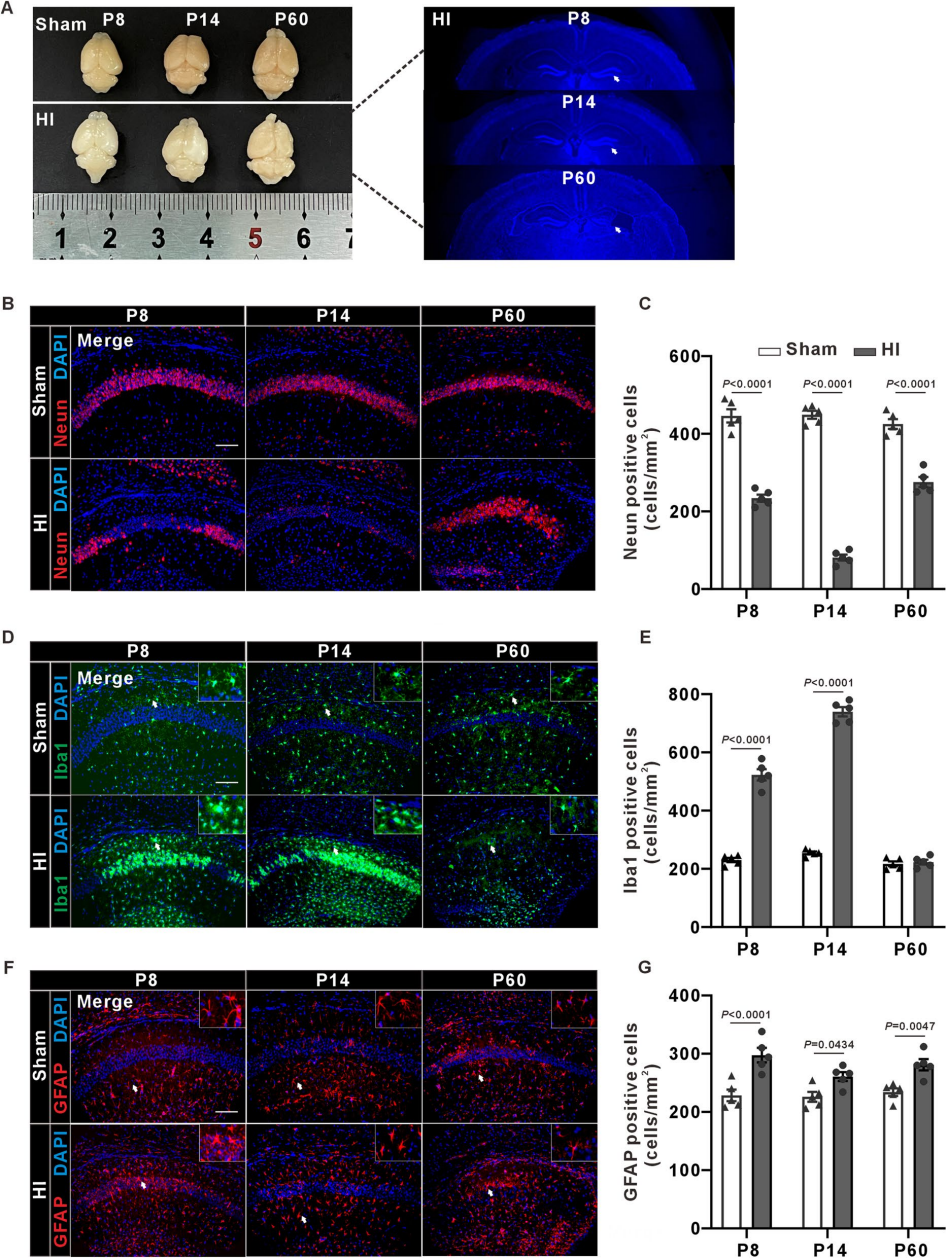
Changes of hippocampal cell numbers in sham and HI mice. **A** Representative gross morphology of mice Brains at P8, P14, and P60 (Left), and the morphological structure of hippocampus in HI mice at P8, P14, and P60 (Right). Blue: DAPI. A noticeable atrophy of the hippocampus on the ligated side in HI mice can be observed at P60. **B** Neun immunofluorescence staining of hippocampal tissue sections, showing a significant reduction of Neun-positive neurons in HI mice. Red: Neun, Blue: DAPI, scale bar = 100 μm. **C** Mean and standard error of the number of Neun-positive neurons (two-tailed unpaired Student’s t-test, n = 5 mice/group). **D** Iba1 immunofluorescence staining, demonstrating a marked elevation in Iba1-positive microglia in HI Mice. Green: Iba1, Blue: DAPI, white arrows: activated microglial cells. **E** The number of Iba1-positive microglia (two-tailed unpaired Student’s t-test, n = 5 mice/group). **F** GFAP immunofluorescence staining, indicating a pronounced increase in GFAP-positive astrocytes in HI Mice. Red: GFAP, Blue: DAPI, white arrows: activated astrocytes. **G** The number of GFAP-positive astrocytes (two-tailed unpaired Student’s t-test, n = 5 mice/group). Cell counting was limited to the hippocampal region, excluding areas such as the cortex. This applies to all quantifications mentioned in panels **C**, **E**, and **G**

To find pathological change of hippocampus in HI mice, we performed immunofluorescence staining and tissue harvest at P8, P14 and P60 (Fig. 1A). The gross morphology of the brain in the HI group was similarly to that in the sham group at P8 and P14, but smaller at P60. The atrophy was especially significant in the right hippocampus (Fig. 2A). The HI group showed a clear decrease of NeuN positive cells compared with the sham group from P8-P14 (Fig. 2B, C). And HI mice exhibited obvious hippocampal atrophy at P60 (Fig. 2B). Iba1-positive microglial cells in the sham group appeared mostly in the resting ramified form with small round cell bodies and thin processes, whereas those in HI group showed considerable branch retraction, thickening, and amoeba-like morphology from P8-P14 (Fig. 2D, E). The size and number of GFAP-positive astrocytes were significantly increased in HI group from P8-P14. HI mice had formed glial scarring at P60 (Fig. 2F, G).

### Differential metabolites and their functional analysis on the sham and HI mice

**Fig.3:**
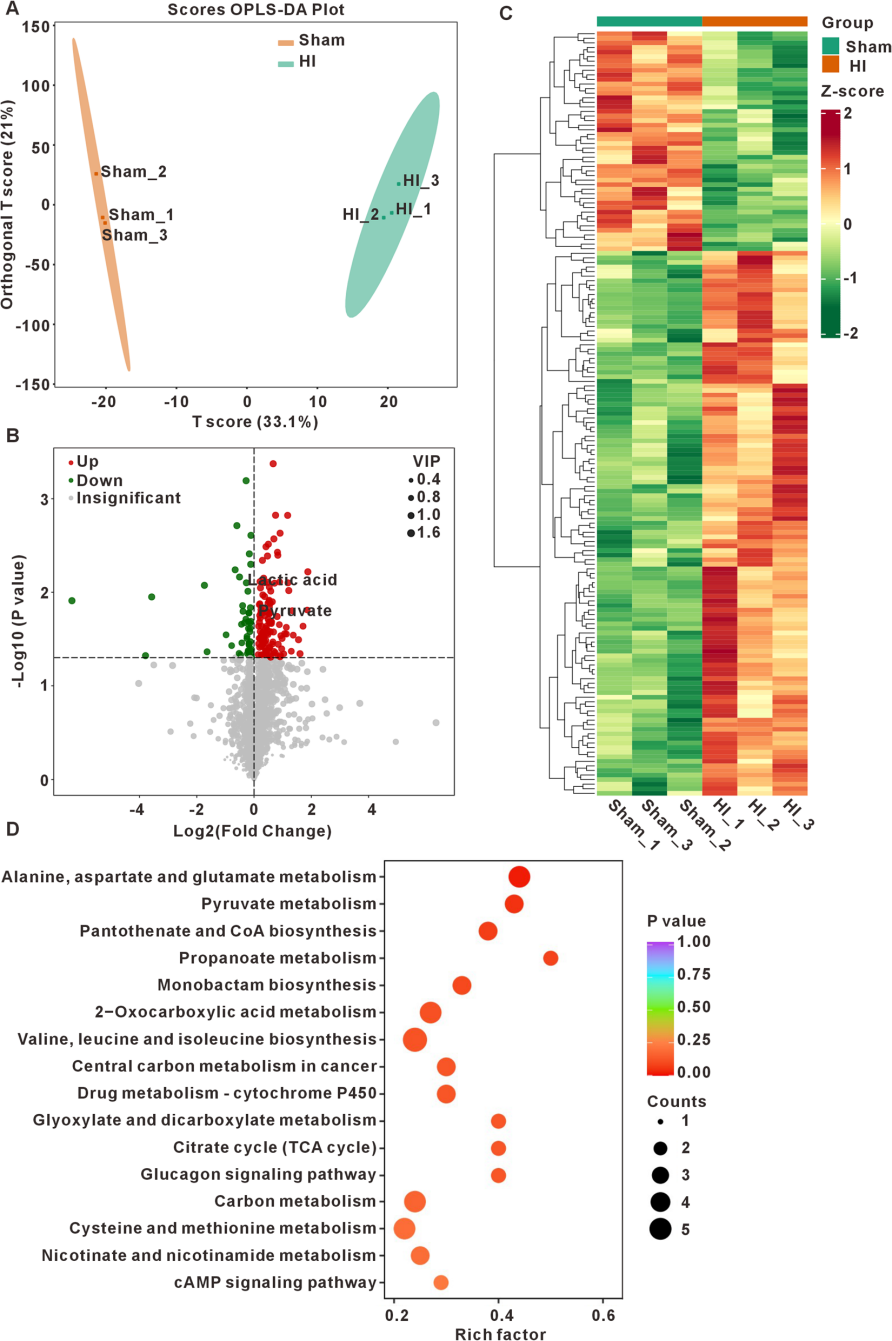
Metabolites and pathways associated with HI. **A** OPLS-DA score plot of sham (green) and HI group (orange). **B** Volcanic diagram of DEMs. Blue denotes down-regulated metabolites(VIP ≥ 1, P < 0.05, FC > 0), red denotes up-regulated metabolites(VIP ≥ 1, P < 0.05, FC < 0) and grey denotes insignificant metabolites. **C** Hierarchical cluster of the DEMs. The left vertical axis shows clusters of DEMs, while the above horizontal axis shows clusters of samples. Red represents high expression and green represents low expression. **D** The bubble chart of KEGG enrichment based on DEMs. The abscissa represents the rich factor corresponding to each pathway, the ordinate represents the pathway name, and the color of the point is the p-value. The redder the point is, the more significant the enrichment is. The size of the dot represents the number of enriched differential metabolites

We analyzed the HI related changes of metabolites in the hippocampus. As depicted in Fig. 3A, The OPLS-DA score plot demonstrates a clear separation between the sham group (orange spots) and the HI group (green spots), suggesting a metabolic distinction between the two conditions. Using VIP value > 1 and p-value < 0.05 as criteria, we identified 48 downregulated and 119 upregulated differential metabolites (DEMs), including pyruvate, lactic acid, succinate, and citric acid (Fig. 3B). More specific information on differential metabolites can be seen in Supplementary Fig. S1. The hierarchical cluster of the DEMs (Fig. 3C) clearly shows metabolite differences between the sham and HI group. KEGG analysis also indicated that the metabolic pathways associated with alanine, aspartate, glutamate and pyruvate were enriched (Fig. 3D).

### Functional analysis of differentially expressed genes between sham and HI mice

**Fig.4:**
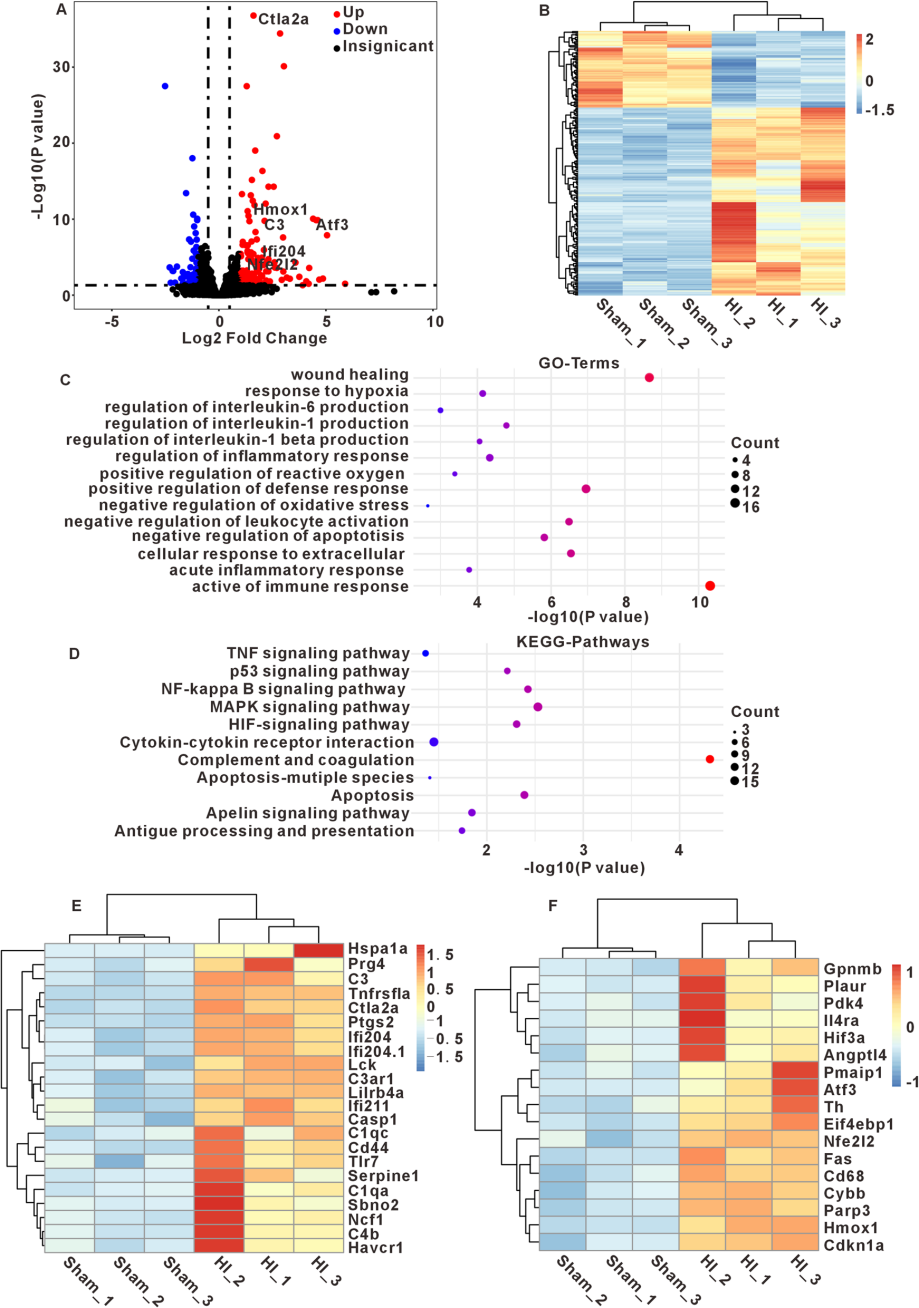
Enrichment analysis of DEGs associated with HI. **A** Volcano map shows DEGs between sham vs HI group. Red: upregulated DEGs (P < 0.05, log2FC > 1); blue: downregulated DEGs (P < 0.05, log2FC < 1). **B** Hierarchical cluster analysis. Different columns in the figure represent different samples, and different rows represent different genes. The colors from blue to yellow indicate gene expression from low to high, respectively. **C**, **D** The bubble chart of GO and KEGG enrichment based on DEGs. The ordinate represents enriched GO functional and KEGG pathway terms of these DEGs, and the abscissa represents the –log 10 (P-values) of the significantly enriched terms. Counts represent the number of DEGs enriched in each GO functional or KEGG pathway term. **E** Heatmap showing the 22 DEGs associated with IL-1, IL-6, immune response and inflammatory response. **F** Heatmap showing the 17 DEGs associated with negative regulation of apoptotic, leukocyte activation and response to hypoxia

RNA-seq analysis revealed 60 downregulated and 147 upregulated differentially expressed genes (DEGs), using criteria of |log2 fold change|> 1 and P < 0.05(Fig. 4A). The differences of gene expression between the sham and HI groups were distinct as depicted by the hierarchical cluster analysis (Fig. 4B). Then, we conducted GO and KEGG analyses to investigate the functional implications of the DEGs. GO analysis showed that the DEGs were associated with oxidative stress and inflammation, including wound healing, activation of immune response, regulation of IL-1, IL-6 and inflammatory et. al. (Fig. 4C). KEGG enrichment analysis revealed that these DEGs were closely related signaling pathways of MAPK, NF-κB, TNF, HIF-1α and apoptosis et. al. (Fig. 4D). At the same time, anti-inflammatory and antioxidant pathways, including cellular response to extracellular stimulus, negative regulation of leukocyte activation and oxidative stress-induced intrinsic apoptotic are also enriched. Figure 4E, F display the expression levels of DEGs associated with oxidative stress/inflammation and antioxidant /anti-inflammatory processes in sham and HI mice. These results suggest that during the acute phase of HI, both oxidative stress/inflammatory responses and anti-inflammatory/antioxidant mechanisms were activated.

### Protective effects of 4OI on HI mice

**Fig. 5:**
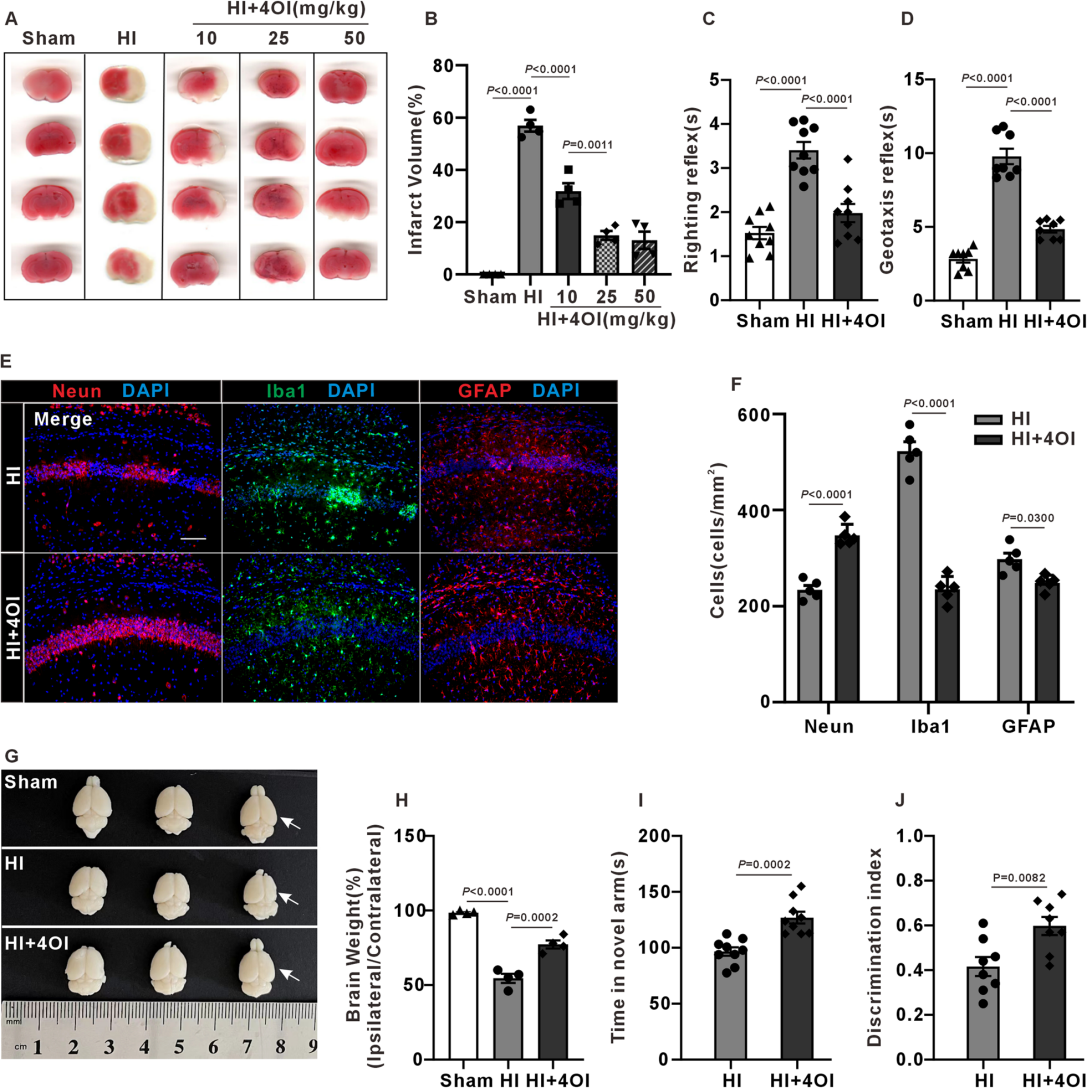
Effect of 4OI treatment on brain histopathological changes and cognitive dysfunction in HI mice. **A** Representative TTC stained coronal brain sections from Sham, HI and HI + 4OI groups 24 h post-HI, it was found that ≥ 25 mg/kg 4OI could significantly reduce the cerebral infarction foci of HI mice. **B** Infarct volume (one-way ANOVA with Tukey’s multiple-comparison test, n = 4 mice/group). **C**, **D** The latency time of righting reflex and geotaxis reflex. Compared with the Sham group, the latency time of HI mice was significantly prolonged, and significantly shortened after 4OI treatment (one-way ANOVA with Tukey’s multiple-comparison test, n = 8 mice/group). **E**, **F** Neun, Iba1, and GFAP immunofluorescence staining of hippocampal tissue sections. Counts of Neun-positive neurons, Iba1-positive microglia and GFAP-positive astrocytes. The 4OI treatment is observed to effectively mitigate the reduction of hippocampal neurons and the proliferation of microglia and astrocytes in HI mice (one-way ANOVA with Tukey’s multiple-comparison test, n = 5 mice/group). scale bar = 100 μm. Cell counting was limited to the hippocampal region, excluding areas such as the cortex**. G**, **H** Comparison of brain morphology and weight in different groups at P60. The right-to-left cerebrum (ipsilateral/contralateral) weight ratio was smaller in HI group, suggesting a significant tissue loss in the ipsilateral hemisphere, which was significantly relieved after 4OI treatment (one-way ANOVA with Tukey’s multiple-comparison test, n = 4 mice/group). **I**, **L** 4OI treatment notably increased exploration time in the Y-maze and discrimination index in the object recognition test (one-way ANOVA with Tukey’s multiple-comparison test, n = 8 mice/group)

Based on the results of omics sequencing, we presumed that 4OI, an anti-oxidant and anti-inflammatory agent, may play a protective role in HI mice. We injected 4OI intraperitoneally with 10, 25 and 50 mg/kg in HI mice after HI. The mean infarct volume significantly decreased from 59.39% to 31.88% in the HI + 4OI (10 mg/kg) group compared to the HI group. A further reduction to 14.92% was seen with 25 mg/kg 4OI. Interestingly, there was no significant difference between the group receiving 50 mg/kg 4OI (average infarct area 14.55%) and the 25 mg/kg 4OI group (Fig. 5A, B). As a result, we chose to use 25 mg/kg 4OI for the subsequent experiments. We performed negative geotaxis and righting reflex tests to evaluate the short-term neurological function at 24 h post HI. HI mice exhibited a prolonged latency in both negative geotaxis and righting reflex tests. Treatment of 4OI significantly reduced the latency in HI mice (Fig. 5C, D). NeuN staining showed that 4OI treatment partially reduced neuronal death, microglia and astrogliosis activation in hippocampal CA1 of HI mice (Fig. 5E, F). In addition, we observed 4OI can also effectively reduce right brain atrophy in adult HI mice (Fig. 5G, H). Moreover, the behavior performance of HI mice in the Y maze and novel object recognition test was significantly improved by 4OI treatment (Fig. 5I, J). Taken together, our results indicate that 4OI treatment effectively alleviate pathological damage in hippocampus and cognitive dysfunction in HI mice.

### 4OI upregulates Nrf-2 expression and exerts anti-inflammatory and antioxidant effects.

**Fig. 6:**
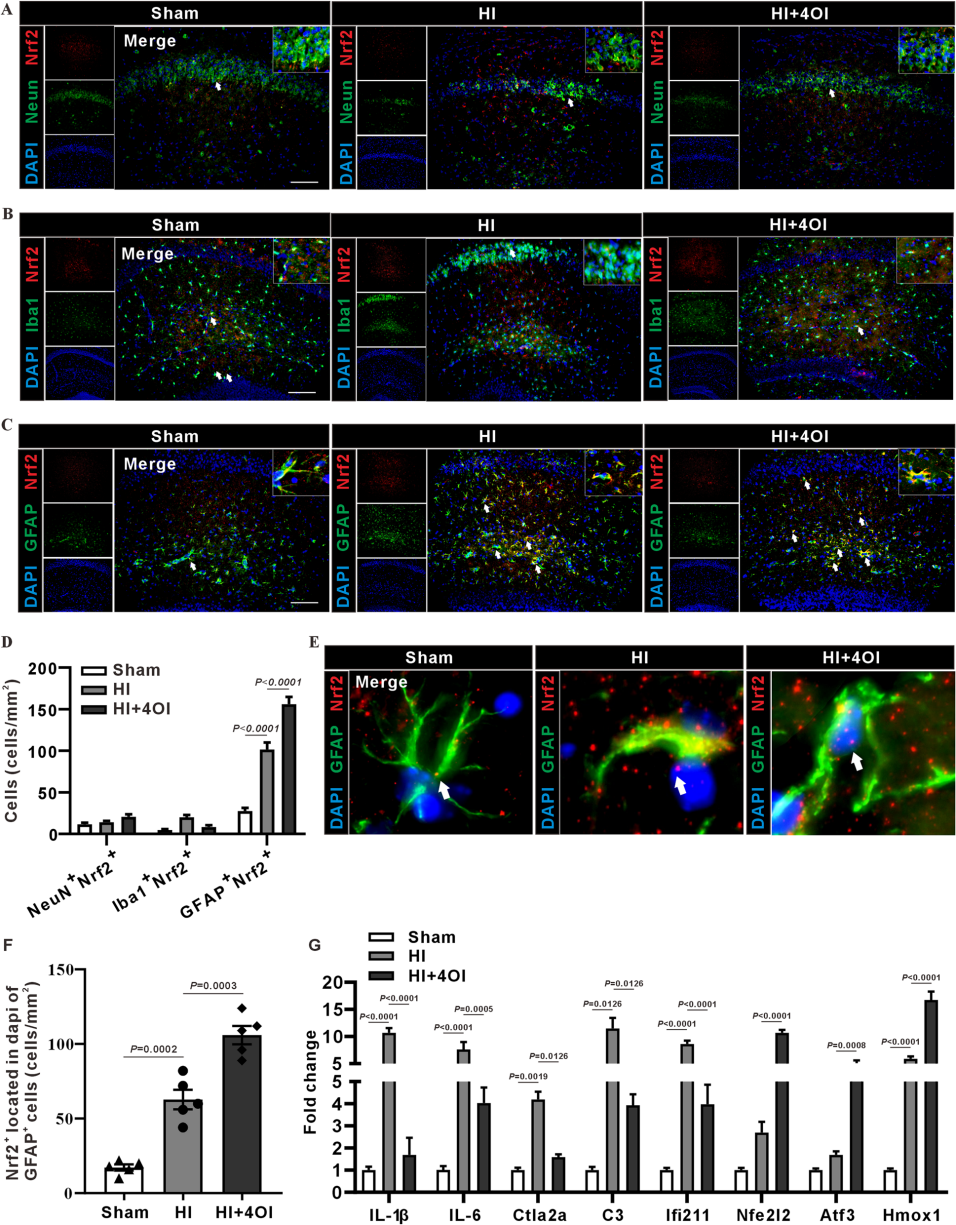
Effects of 4OI on Nrf2 expression, inflammatory cytokines and antioxidant enzymes. **A-C** Immunofluorescence staining of Nrf2 with NeuN, Iba1, and GFAP in the mouse hippocampus. These images demonstrate a higher colocalization of Nrf2 with GFAP and a comparatively lower colocalization with NeuN and Iba1 (n = 5 mice/group). Cell counting was limited to the hippocampal region, excluding areas such as the cortex**. D** Statistical graph of the count of Nrf2-positive cells (one-way ANOVA with Tukey’s multiple-comparison test, n = 4 mice/group). The proportion of Nrf2^+^GFAP^+^ cells is higher in the HI group compared to the Sham group, and even higher in the HI + 4OI group. The proportion of Nrf2^+^ cells is lower in both NeuN^+^ and Iba1^+^ cells across all groups. **E** Representative plot illustrating the spatial relationship between Nrf2 and DAPI in GFAP^+^ cells. Lesser colocalization of Nrf2 with DAPI is observed in both the Sham and HI groups, with an increased colocalization in the HI + 4OI group. scale bar = 100 μm. **F** Statistical plot of the count of GFAP^+^ cells with Nrf2 expression in the nucleus (one-way ANOVA with Tukey’s multiple-comparison test, n = 4 mice/group). **G** Quantitative analysis of IL-1β, IL-6, C3, Ctla2a, Ifi211, Nfel2l2, ATF3, HO-1 and Atf3 mRNA levels in the hippocampus of mice 24 h post-HI using RT-PCR. The 4OI treatment significantly reduced the expression levels of IL-1β, IL-6, C3, Ctla2a and Ifi211while it enhanced the expression of Nfel2l2, HO-1 and ATF3 in the HI mice (two-way ANOVA with Tukey’s multiple-comparison test, n = 3 mice/group)

Some studies showed that 4OI can promote the dissociation of Nrf2 and Keap1, Nrf2 nuclear translocation and Nrf2-dependent gene expression [27, 40]. Our immunofluorescence staining for NeuN (Fig. 6A), Iba1(Fig. 6B) and GFAP (Fig. 6C) indicated a clear co-localization of astrocytes with Nrf2 in the hippocampus of HI mice. After treatment with 4OI, reactive astrocytes exhibited a decrease in cell body size, indicative of a reversion to a quiescent state, and there was a significant increase in the number of Nrf2^+^GFAP^+^ cells. These results suggest that 4OI not only suppresses the activation of astrocytes but also enhances their Nrf2 expression (Fig. 6D). Figure 6E illustrates the colocalization of Nrf2 with the nucleus in astrocytes, showing that the HI group had less Nrf2 within nucleus, while 4OI treatment significantly increased the number of Nrf2 in the nucleus (Fig. 6F). Therefore, 4OI has the effect of increasing the level of Nrf2 and promoting its nuclear translocation. RT-PCR results revealed a substantial elevation in the expression levels of IL-1β, IL-6, C3, Ctla2a and Ifi211 in the HI group compared to the sham group. Notably, this increase was effectively mitigated by the treatment of 4OI (Fig. 6G, H). In addition, the Nfe2l2, HO-1 and Atf3 expression levels were elevated in the HI group and further increased with 4OI treatment (Fig. 6I, J). These results suggest that 4OI may inhibit the production of pro-inflammatory factors by promoting the expression of antioxidant enzymes.

### The effects of 4OI on Nrf2 in astrocytes and microglia

Previous studies on the inflammatory models induced by LPS have shown that 4OI primarily exerts its anti-inflammatory effects on microglial/macrophage cells [31, 41]. Our in vivo studies on the HI model also confirmed the anti-inflammatory and antioxidative effects of 4OI. However, our results suggest that 4OI may exert its effects by promoting the nuclear translocation of Nrf2 in astrocytes, without significantly affecting Nrf2 expression and nuclear translocation in microglial cells. To further confirm the cellular targets of 4OI, we established an in vitro OGD/R model to examine the effects of 4OI on Nrf2 expression and nuclear translocation in astrocytes (CB-D1A) and microglia (BV2).

**Fig. 7:**
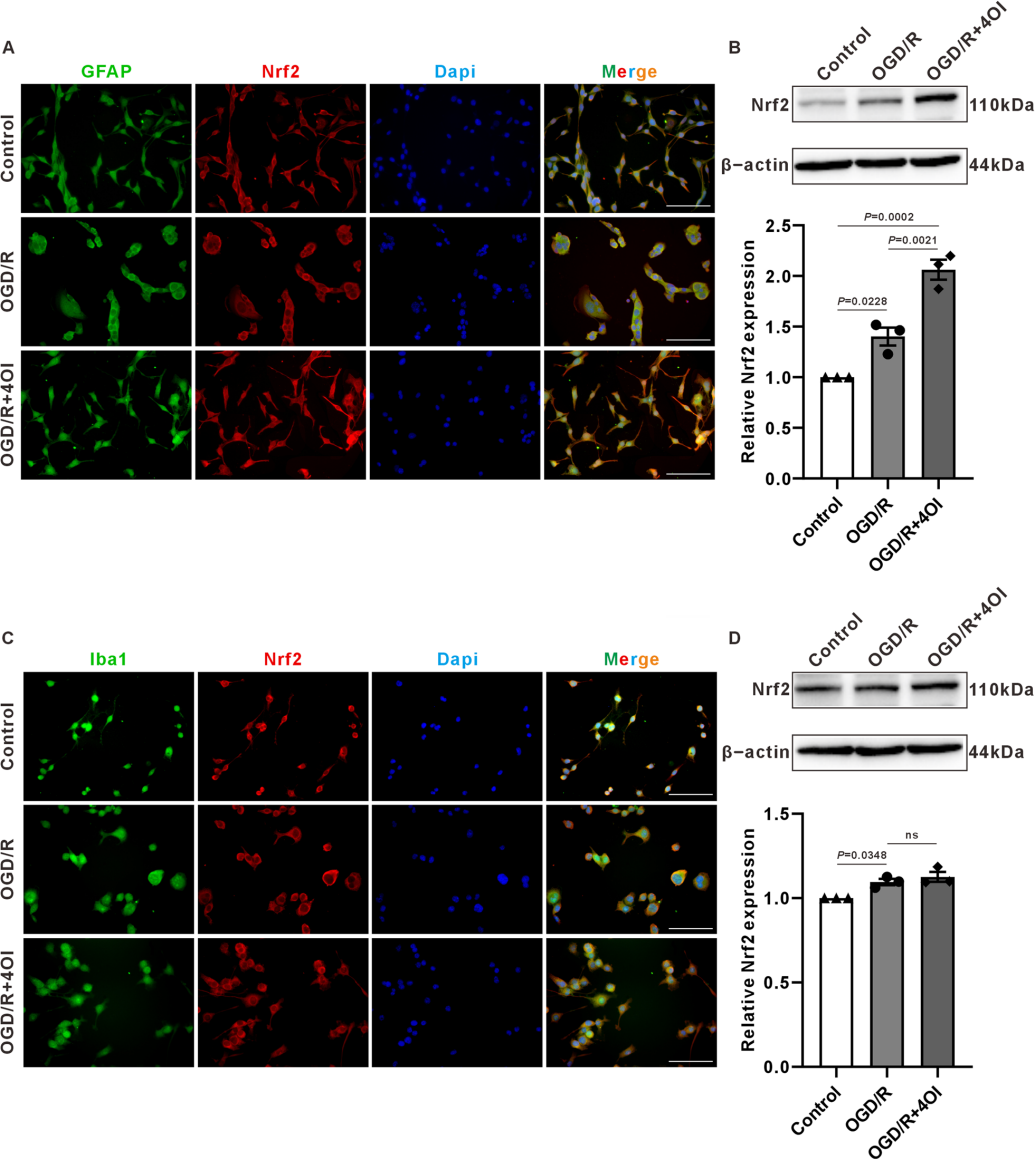
The effect of 4OI on Nrf2 expression levels in BV2 and C8-D1A cells. **A** Immunofluorescence staining of Nrf2 in C8-D1A cells. It can be observed that the expression of Nrf2 in the cytoplasm of the control group and the OGD/R group was more than that in the nucleus, while in the OGD/R + 4OI group, there is no significant difference in Nrf2 expression between the cytoplasm and nucleus. scale bar = 100 μm. **B**, **C** Representative picture of Western blot data from C8-D1A astrocytes. The quantitative protein analysis graph showed that 4OI significantly increases the expression level of Nrf2 (one-way ANOVA with Tukey’s multiple-comparison test, n = 3 /group). **C** Immunofluorescence staining schematic of Nrf2 in BV2 cells. In all three groups, Nrf2 expression is higher in the cytoplasm than in the nucleus. scale bar = 100 μm. **D**, **E** Representative picture of Western blot data from BV2 microglia. The quantitative protein analysis graph showed no significant difference in Nrf2 expression levels among the three groups (one-way ANOVA with Tukey’s multiple-comparison test, n = 3 /group)

Nrf2 was significantly expressed in the cytoplasm of CB-D1A cells in both control and OGD/R groups, with lesser expression in the nucleus. However, in the OGD/R + 4OI group, a noticeable increase in nuclear Nrf2 was observed. In BV2 cells, Nrf2 expression was limited to the cytoplasm in control, OGD/R and OGD/R + 4OI groups. Moreover, Western blot results further confirmed that 4OI increased the expression of Nrf2 in astrocytes, while it had no significant effect on Nrf2 expression in BV2 cells. These findings suggest that 4OI primarily enhances Nrf2 expression and nuclear translocation in astrocytes (Fig. 7).

### Silencing the Nrf2 of astrocytes can attenuate the anti-inflammatory and antioxidant effects of 4OI

**Fig. 8:**
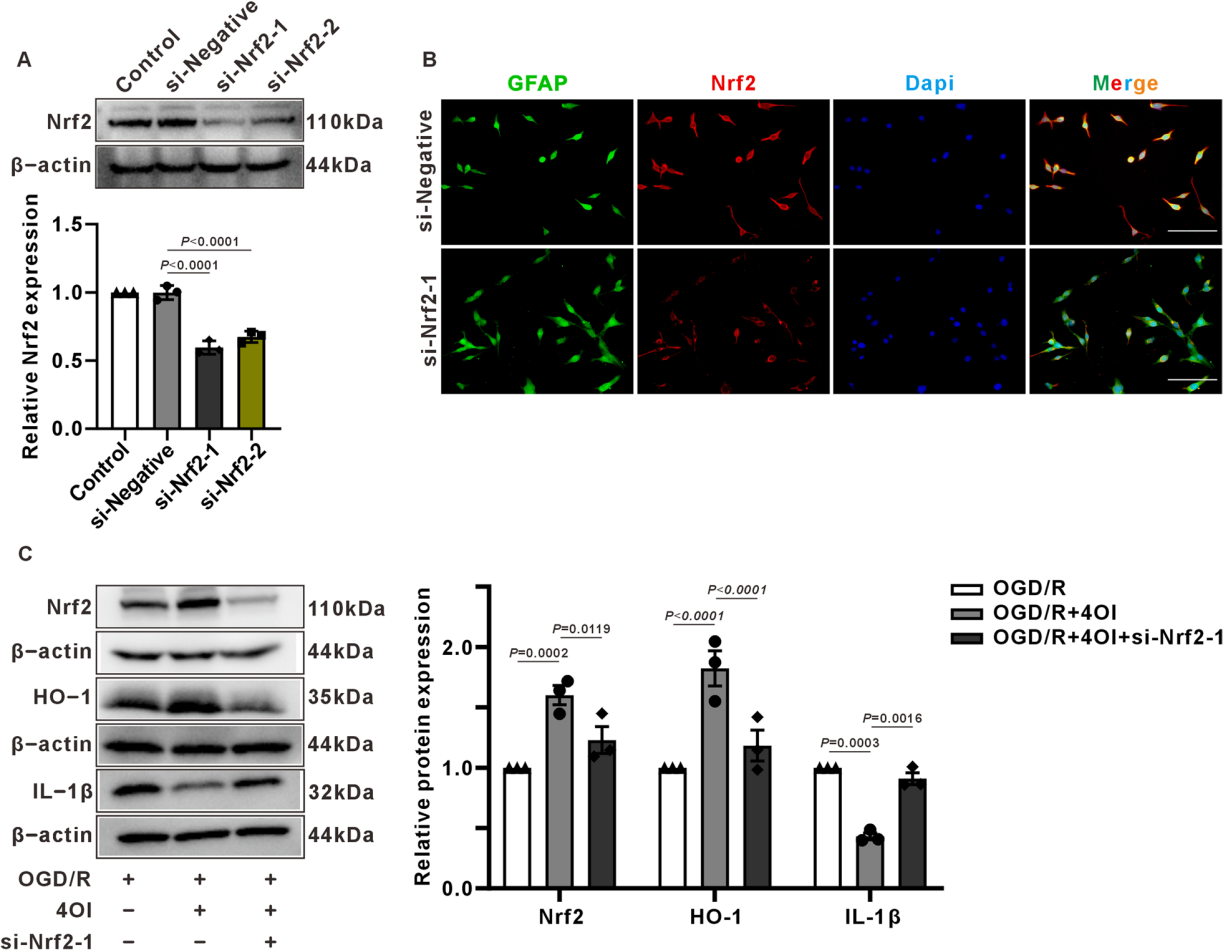
Effect of Nrf2 on HO-1 and IL-1β expression in silencing C8-D1A cells. **A** Western blot data and protein quantification charts of Nrf2 protein in C8-D1A cells in the Control, si-Negative and si-Nrf2 groups showed that the expression of Nrf2 protein in the si-Nrf2-1 and si-Nrf2-2 groups was significantly reduced (one-way ANOVA with Tukey’s multiple-comparison test, n = 3 /group). **B** Immunofluorescence staining of Nrf2 expression in C8-D1A cells after Nrf2 siRNA transfection, showing that the immunofluorescence intensity of Nrf2 in the si-Nrf2-1 group decreased. scale bar = 100 μm. **C** Western blot data and protein quantification charts of Nrf2, IL-1β and HO-1 in C8-D1A cells with OGD/R, OGD/R + 4OI and OGD/R + 4OI + si-Nrf2-1 groups (two-way ANOVA with Tukey’s multiple-comparison test, n = 3 /group). In the si-Nrf2-1 + OGD/R + 4OI group, transfection of Nrf2 siRNA inhibited the effect of 4OI on the promotion of Nrf2 and HO-1 expression and the inhibition of IL-1β expression

To validate specific 4OI protection, we used the Nrf2 siRNA constructs to reduce the expression of Nrf2 in C8-D1A cells, and then exanimated the effect of 4OI in rectifying the abnormal expression of inflammatory factors and antioxidant enzymes induced by OGD/R. Western blot analysis confirmed successful transfection of both Nrf2-1 siRNA and Nrf2-2 siRNA constructs, effectively reducing Nrf2 protein levels in C8-D1A cells (Fig. 8A). As both si-Nrf2-1 and si-Nrf2-2 showed similar efficacy in suppressing Nrf2 protein, Nrf2-1 siRNA was chosen for subsequent experiments. Immunofluorescence staining further validated the reduction in Nrf2 fluorescence intensity in C8-D1A cells transfected with Nrf2-1 siRNA plasmid (Fig. 8B). Subsequent analysis revealed that 4OI treatment significantly upregulated Nrf2 and HO-1 expression while downregulating IL-1β levels after OGD/R in normal C8-D1A cells. However, these effects were attenuated in Nrf2-1 siRNA-transfected C8-D1A cells, indicating that 4OI mediates its anti-inflammatory and antioxidative effects through Nrf2 in astrocytes.

## Discussion

In this study, we firstly employed an approach combining untargeted metabolomics with transcriptomics to screen for changes in metabolites and gene expression in a mouse model of acute HI. The results revealed a significant accumulation of lactate and succinate, as well as elevated expression of inflammatory genes related to the IFN and NF-κB pathways. Additionally, we observed increased expression of antioxidant stress genes, such as Atf3, Nfe2l2, and Hmox-1. Furthermore, intraperitoneal injection of 4OI in HI mice reduced the infarct area, suppressed the proliferation of microglia and astrocytes, and mitigated neuronal death, resulting in improved cognitive function in adult HI mice. The neuroprotective effects of 4OI were potentially mediated through upregulation of Nrf2 levels in astrocytes, inhibition of IL-1β and IL-6 production, and increased expression of HO-1 and Atf3. Our findings contribute to a better understanding of the complex pathophysiological mechanisms underlying HIE and provide insights for the development of targeted therapeutic interventions.

Clinical researchers have found that HIE patients often exhibit significant cognitive impairments in adulthood, including difficulties in learning, memory issues, and attention deficits [42–45]. The damage to hippocampal neurons caused by ischemia and hypoxia is likely a key pathological process in this context. In our study, we observed pronounced neuronal death in the CA1 region of HI mice at P8, with neuronal deaths in the CA1 region further increasing over time without intervention. Concurrently, there was a significant proliferation and activation of microglia and astrocytes in the CA1 area. By P60, the ipsilateral hippocampal region in the HI mice showed atrophy and astroglial scar. Neurons in the hippocampal CA1 region have been proven to play a crucial role in cognition, spatial learning, and memory processes [46, 47]. Related studies have already demonstrated memory impairment in the HI model [48, 49]. Similarly, in our study, adult HI mice exhibited a marked reduction in exploration time of novel arms and objects in the Y-maze and novel object recognition tests. These findings further confirm the central role of hippocampal neuronal damage in cognitive dysfunction caused by HIE.

Previous studies indicated that the initial pathological process (0–6 h) of HIE is characterized by an energy deficit. Hypoxia compels cells to transition from the tricarboxylic acid (TCA) cycle to anaerobic glycolysis as a compensatory energy source, leading to an increased production of pyruvate and lactate. High levels of lactate and pyruvate can increase the production of reactive oxygen species (ROS) and inflammatory mediators, damage the blood–brain barrier (BBB), and lead to edema. These changes eventually result in sustained neuronal damage within 6–48 h post-injury [13, 21, 50, 51]. Consistent with this, we also observed elevated levels of pyruvate and lactate in the hippocampal tissue of acute HI mice, indicating a greater reliance on glycolysis by the cells. Our results of high-throughput transcriptomics revealed upregulated expression of genes related to oxidative stress and inflammation. Further analyses confirmed that these gene expression changes are associated with biological processes related to IFN and IL-1β/6, and the KEGG pathways of NF-κB, apoptosis, HIF-1α. This suggests that oxidative stress and inflammatory responses may be the primary factors contributing to neuronal damage in the hippocampus.

On the other hand, concomitant with the occurrence of oxidative stress and inflammatory responses, anti-inflammatory and antioxidant mechanisms are also triggered. One of a key molecule of endogenous antioxidant and anti-inflammatory responses is Nrf2. Under oxidative stress, Nrf2 dissociates from Kelch-like ECH-associated protein 1 (Keap1) and translocates to the nucleus, where it binds to antioxidant response elements (AREs) and upregulates the expression of various antioxidant genes, notably HO-1 [52]. Additionally, Nrf2 inhibits the expression of pro-inflammatory cytokines, such as IL-1β and IL-6, by modulating the Atf3/IκBζ pathway [53–56]. Our study also found that the expressions of Nfe2l2, Hmox1, and Atf3 were elevated in the hippocampus of HI mice, suggesting that the mechanisms of anti-inflammatory and antioxidant are triggered during the early stages of HI. These results imply that enhancing the antioxidant and anti-inflammatory response mediated by Nrf2 may play a neuro-protective role in HI. Furthermore, our immunofluorescence staining results revealed that the expression level of Nrf2 significantly increased in HI mice, and Nrf2 predominantly co-localizes with astrocytes, indicating that HI predominantly triggers Nrf2 expression in astrocytes. Previous study has also reported that multiple Nrf2-targeted antioxidant and detoxification genes are preferentially expressed in glial cells [57, 58]. Indeed, the protective role of Nrf2 in astrocytes has been shown in mouse models of ALS, Parkinson’s disease, cerebral hypoperfusion and Alzheimer’s disease with the astrocyte-specific Nrf2 transgene [59]. Thus, astrocytes may be the primary cell type where Nrf2 exerts its antioxidative and anti-inflammatory effects.

Recent studies have shown that 4OI, a derivative of itaconate with high cell permeability, is one of a valid Nrf2 activator. It facilitates the dissociation of Keap1 and Nrf2, reducing the ubiquitination of Nrf2 and promoting its nuclear translocation to regulate the expression of downstream genes related to antioxidant and anti-inflammatory responses [60]. In vitro study shows that 4OI can protect SH-SY5Y cells and epigenetically de-repressed primary neurons from H_2_O_2_ [61]. In vivo experiments have also confirmed that 4OI can alleviate β_2_M-induced cognitive dysfunction and hippocampal neurogenesis impairment [62]. For the first time, we examined the effects of 4OI as a therapeutic agent for HIE. Our results show that 4OI effectively reduces the infarct area in HI mice, inhibits the activation and proliferation of microglia and astrocytes, and decreases neuronal apoptosis. Furthermore, 4OI significantly alleviates brain atrophy in adult HI mice and mitigates their cognitive dysfunction. In HI mice, the expression level of Nrf2 in astrocytes mainly increased in the cytoplasm. However, 4OI treatment increased the expression level of Nrf2 in both cytoplasm and nucleus. In addition, our in vitro experiments have demonstrated that 4OI can promote Nrf2 expression and nuclear transfer in astrocytes. Simultaneously, the levels of Nfel2l2, HO-1 and Atf3 are significantly increased in the 4OI treatment group, and the levels of IL-1β, IL-6, C3, Ctla2a and Ifi211 are significantly decreased. In addition, after knocking down the expression of Nrf2 in astrocytes cultured in vitro with the Nrf2 siRNA plasmid, we found that the anti-inflammatory and antioxidant effect of 4OI against OGD/R was significantly diminished, suggesting that 4OI exerts anti-inflammatory and antioxidant effects through Nrf2 in astrocytes.

There are also some shortcomings in this study. First, the sample size of the included studies in this experiment was small, which did not fully validate the safety and efficacy of 4OI as a therapeutic drug. Secondly, this study did not involve clinical data and could not confirm the actual therapeutic effect of 4OI on HIE patients. Finally, our in vivo experiments used Bulk-RNA sequencing and non-targeted metabolome sequencing, and the results did not determine the specific cell type. Although our results from in vivo immunofluorescence staining and in vitro cell culture experiments suggest that 4OI can regulate Nrf2 expression and function in astrocytes, it cannot be ruled out that 4OI also plays a similar role in other cell types.

In conclusion, we found that in the acute phase of HI, there is an accumulation of pyruvate and lactate in the hippocampal tissue, accompanied by oxidative stress and pro-inflammatory, as well as increased expression of antioxidative stress and anti-inflammatory genes. We also confirmed that 4OI exerts neuroprotective effects against HI through the Nrf2 pathway in astrocytes. It is important to note that our experimental observations have focused on the early stages of HI, and it remains unclear whether Nrf2 plays a similar role in other cell types in the late HI stages. This issue deserves further study. Nevertheless, our research contributes to a more comprehensive understanding the complex pathophysiology of HIE and provides new insights for targeted therapeutic interventions.

### Supplementary Information

Supplementary Material 1 (PDF 45 KB)

## Data Availability

Data supporting the findings of this study are available upon reasonable request to the corresponding author.

## Abbreviations

HIE: Hypoxic ischemic encephalopathy
HI: Hypoxic ischemic
4OI: 4-Octyl itaconate
Nrf2: Nuclear factor erythroid-2-related factor
HO-1: Hmox1
TTC: 2,3,5-Triphenyltetrazolium chloride
