# Supplementary figures and images for: Protecting effects of 4-octyl itaconate on neonatal hypoxic-ischemic encephalopathy via Nrf2 pathway in astrocytes
Source: J Neuroinflammation. 2024 May 17;21:132. doi: 10.1186/s12974-024-03121-8 (PMC11102208; doi:10.1186/s12974-024-03121-8)

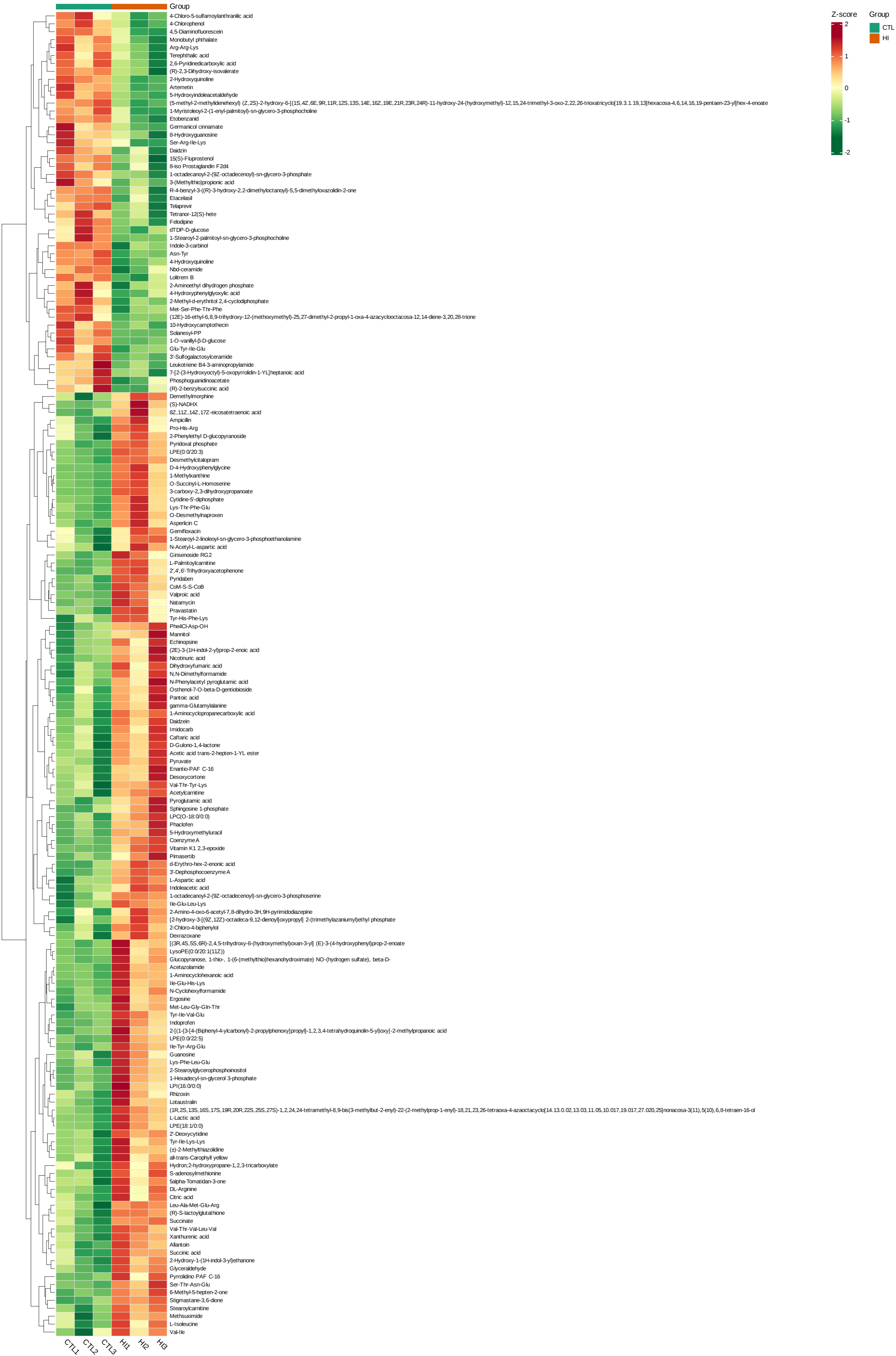

Supplement: Supplementary file 1 — Supplementary Material 1 (PDF 45 KB) [file 12974_2024_3121_MOESM1_ESM.pdf]
